# Hospital sustainability indicators and reduction of socio-environmental impacts: a scoping review

**DOI:** 10.1590/1980-220X-REEUSP-2022-0364en

**Published:** 2023-06-23

**Authors:** Daniela Menezes Galvão, Marta Regina Cezar-Vaz, Daiani Modernel Xavier, José Gustavo Monteiro Penha, Luciano Garcia Lourenção

**Affiliations:** 1 Universidade Federal do Rio Grande, Escola de Enfermagem, Rio Grande, RS, Brazil.

**Keywords:** Conservation of Natural Resource, Environment, Sustainable Development, Hospital, Sustainable Development Indicator, Conservación de los Recursos Naturales, Ambiente, Desarrollo Sostenible, Hospitales, Indicadores de Desarrollo Sostenible, Conservação dos Recursos Naturais, Meio Ambiente, Desenvolvimento Sustentável, Hospitais, Indicadores de Desenvolvimento Sustentável

## Abstract

**Objective:**

To synthesize knowledge about hospital sustainability indicators and evidence of reduced socio-environmental impact.

**Method:**

Literature scoping review using Pubmed, Science Direct, Scielo and Lilacs databases. Studies in a time frame of 10 years, addressing hospital sustainability indicators and evidence of reduced socio-environmental impact published in any language were included.

**Results:**

A total of 28 articles were included, most were applied research, published in 2012, in English. Studies showed ways to save water and energy, as well as ways to monitor and mitigate the impact of activities related to effluents, waste and emissions. All studies had nursing work directly or indirectly involved in hospital sustainability.

**Conclusion:**

The possibilities of generating less impact on the environment and increasing the economy/efficiency of a hospital are countless. The particularities of each hospital must be taken into account and workers, especially nurses, should be involved.

## RESUMO

**Objetivo:**

Sintetizar o conhecimento acerca dos indicadores de sustentabilidade hospitalar e evidências de redução do impacto socioambiental.

**Método:**

Revisão de literatura, do tipo *scoping review*, utilizando as bases de dados *Pubmed*, *Science Direct*, *Scielo* e *Lilacs*. Foram incluídos estudos com recorte temporal de 10 anos, publicados em qualquer idioma, que abordassem indicadores de sustentabilidade hospitalar e evidências de redução do impacto socioambiental.

**Resultados:**

Um total de 28 artigos foram incluídos, a maioria teve como tipo de estudo a pesquisa aplicada, publicados em 2012, no idioma inglês. Os estudos demonstraram maneiras de economizar água e energia, bem como, formas de monitorar e mitigar o impacto de atividades relacionadas a efluentes, resíduos e emissões. Todos os estudos tinham o trabalho da enfermagem envolvido de forma direta ou indireta na sustentabilidade hospitalar.

**Conclusão:**

As possibilidades de gerar menos impacto ao meio ambiente e aumentar a economia/eficiência de um hospital são inúmeras. Há de se levar em consideração as particularidades de cada hospital e envolver os trabalhadores, em especial a enfermagem.

## RESUMEN

**Objetivo:**

Sintetizar conocimientos sobre indicadores de sostenibilidad hospitalaria y evidencias de reducción del impacto socioambiental.

**Método:**

Revisión de literatura, scoping review, utilizando las bases de datos Pubmed, Science Direct, Scielo y Lilacs. Se incluyeron estudios con un horizonte temporal de 10 años, publicados en cualquier idioma, que abordaran indicadores de sostenibilidad hospitalaria y evidencias de reducción del impacto socioambiental.

**Resultados:**

Se incluyeron un total de 28 artículos, la mayoría de investigación aplicada, publicados en 2012, en inglés. Los estudios mostraron formas de ahorrar agua y energía, así como formas de monitorear y mitigar el impacto de las actividades relacionadas con efluentes, desechos y emisiones. Todos los estudios tenían trabajo de enfermería involucrado directa o indirectamente en la sostenibilidad del hospital. **Conclusión:** Las posibilidades de generar un menor impacto en el medio ambiente y aumentar la economía/eficiencia de un hospital son innumerables. Hay que tener en cuenta las particularidades de cada hospital y se debe involucrar a los trabajadores, especialmente a los enfermeros.

## INTRODUCTION

Climate change is one of the greatest health hazards of the 21^st^ century. It is affecting the health of many people, and causing deaths from diseases related to extreme weather events such as storms, heat waves and floods, vector-borne diseases, damage to food systems, among others^(1)^. Given this scenario, all industries need to develop strategies to reduce the emission of greenhouse gases (GHG) and other pollutants^(2)^. There is a growing interest in issues related to environmental sustainability in companies, especially in hospitals, which are considered major polluters of the environment and this reflects on the quality of life and health of the population^(3)^.

Health services in the United Kingdom, for example, are responsible for 3.5% of the total GHG emitted in the world and more than half of these are indirectly caused by the consumption of pharmaceutical products and medical devices, while in the United States, these same services represent 10% of GHG emissions and other atmospheric pollutants^(4)^.

Therefore, health professionals around the world need to have more knowledge and be aware of their work, the excessive expenditure on materials and implications of climate change on public health^(5)^. Environmental pollution is leading to an increase in average temperatures, sea level rise, drastic changes in weather events, worsening air quality, exposure to heat-related morbidity and mortality, increased cases of skin cancer, in addition to harmful effects on mental health^(6)^.

Workers on the front line of this health crisis have been challenged by new pathologies and need to respond to new health needs of the population^(7)^, such as optimally meeting the needs of users, complying with legal requirements, keeping the organization sustainable and causing the least amount of impacts to the system. Managers use several indicators to better understand these requirements and know the reality of their institution, but not only that. It is necessary to spread the concepts, practices and sustainable actions in the routine of all sectors in the organization^(8)^.

The term indicator means estimating, showing, pointing, and can be applied in different scenarios^(9)^. Sustainability indicators began to be used as measurement tools after the Rio-92 conference with the aim to monitor, evaluate and measure the current situation of a given society and based on the analysis, propose actions for the promotion of sustainability through reflections between what was planned and executed^(10)^.

Although there are no indicators currently assessing the sustainability of hospitals in a multidimensional way, some studies suggest the use of indicators from the perspective of five dimensions: strategic, economic, social, environmental and technical, and the environmental dimension is the focus of this review^(8)^. In this regard, many companies use the guidelines of the Global Reporting Initiative (GRI) (an independent international organization that helps companies to report their impacts through sustainability reports) to communicate their environmental impacts. These are generally divided into two groups: the first is aimed at optimization of resources (energy and water) and the second at monitoring and mitigating the impact of its activities (effluents, waste and emissions)^(11)^.

The development of this review is necessary given the few studies on hospital sustainability indicators, as well as the scarcity of research on the subject authored by nurses, even though these studies are directly or indirectly related to nursing work.

The relevance of this theme lies in the fact that from it, managers will be able to know the reality of the place where they work and make the best decisions based on the evidence of socio-environmental impacts published in the studies and later adjust their reality. In addition to allowing the deepening of knowledge about hospital sustainability, it can promote more environmental awareness to the nursing team, since they are present in most hospital activities and have a relevant role in terms of sustainability in this scenario. This scoping review will allow the systematic and summarized grouping of the current status of studies directed at hospital sustainability indicators.

The main question of the present study is: *which studies deal with indicators of hospital sustainability and evidence of reduced socio-environmental impact?* Hypotheses are national and international studies that refer water, energy and solid waste expenditure as indicators, having as evidence: savings with the use of pedal-operated faucets, use of solar panels and waste recycling.

The aim is to synthesize knowledge about hospital sustainability indicators and evidence of reduced socio-environmental impact.

## METHODS

This is a literature scoping review study based on the theoretical framework proposed and developed by the Joanna Briggs Institute (JBI)^(12)^. This review was conducted and reported in accordance with the assumptions of the Preferred Reporting Items for Systematic reviews and Meta-Analyses extension or Scoping Reviews (PRISMA-ScR)^(13)^.

The scoping review is well suited to this study as it is not intended to assess the quality of available evidence, but rather to obtain a representative view of studies. The study was developed according to the following steps: development of the research question (s); identification of relevant studies; selection of studies; data extraction; synthesis and grouping of results; and disclosure^(12)^.

### Identification of the Research Question

The guiding question of this review was developed using the PCC strategy – population (P), concept (C), context (C) – to define the criteria for selecting articles, with (P) as hospital indicators; (C) socio-environmental sustainability and (C), hospital environment. The guiding question defined using this mnemonic combination was ‘Which studies deal with hospital sustainability indicators and evidence of reduced socio-environmental impact?’

Before starting the development of this study, searches were carried out on the Open Science Framework, Database of Abstracts of Reviews of Effects (DARE), The Cochrane Library and the International Prospective Register of Ongoing Systematic Reviews (PROSPERO) sites, in order to identify research of similar reviews and avoid duplication of studies. As no similar studies were found, this review was registered in the Open Science Framework (OSF) under protocol number osf.io/5u7f6.

**Chart 1.**
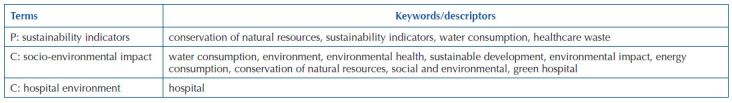
Descriptors or keywords identified and in line with components of the research question according to the PCC strategy – Rio Grande, RS, Brazil, 2022.

Using the PCC acronym words, searches for articles related to the topic were performed in Google academic and the more prevalent descriptors in these articles were observed. Then, these descriptors were selected for further research in databases, as shown in Chart 1.

### Information Sources and Inclusion and Exclusion Criteria

Inclusion and exclusion criteria for each element of the PCC acronym were outlined as follows: Population (P), all studies involving hospital indicators of the environmental dimension were considered. Studies dealing with other dimensions (strategic, economic, social and technical) were excluded; Concept (C), all studies focusing on socio-environmental sustainability were considered, and studies referring to other concepts of sustainability (economic, public and private law, for example) were excluded; Context (C), all studies related to the hospital environment were considered. Studies referring to other contexts (community outpatient clinics, home care services, for example) were excluded.

The criteria defined for the selection of databases (Public Medical Literature Analysis and Retrieval System Online (PubMed); Science Direct, Elsevier database; Scientific Electronic Library Online (SciELO); Latin American and Caribbean Health Sciences Literature (LILACS) was the availability of articles for consultation through search engines with support of Boolean descriptors and operators, as these are up-to- date databases. Studies published in English were selected, as it is considered the preferred language for scientific articles in the health area. However, relevant studies in other languages were also considered. After this stage, the references of all included articles were reviewed to identify other studies that could also meet the selection criteria.

General inclusion criteria comprised articles in all languages published in the last 10 years, as they are more recent studies. General exclusion criteria were incomplete articles, articles not available in full and gray literature (theses and dissertations, conference proceedings, reports, government documents, among others). Note that gray literature was not prioritized in view of its various publication interests (theses and dissertations, conference proceedings, reports, government documents, among others), in addition to scientific literature corresponding to the unitary focus of the present study. Even though theses and dissertations, for example, are in academic and therefore scientific contexts, they were not included, given the understanding that the level of academic and scientific equity is reached in the publication of articles in peer-reviewed journals.

### Search Strategies

**Chart 2:**
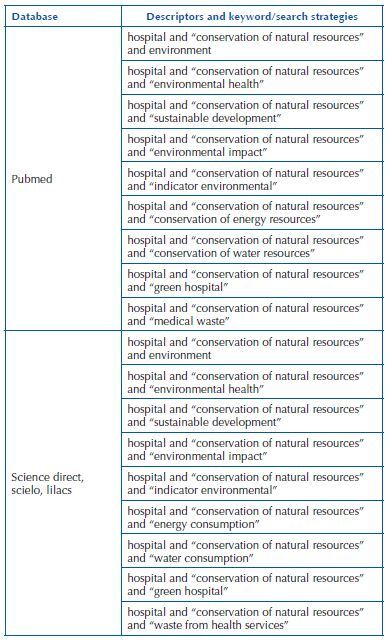
Databases, descriptors and search strategies – Rio Grande, RS, Brazil, 2022.

The electronic search was performed from April to July 2022, using health sciences descriptors (DECS) in English, or medical subject headings (MESH) for searches in the Public Medical Literature Analysis and Retrieval System Online (PubMed), together with the Boolean operator ‘AND’ and quotation marks in compound terms. The search for scientific production was performed in journals indexed in databases through the portal of the Coordination for the Improvement of Higher Education Personnel (Portuguese acronym: CAPES) and the Virtual Health Library (VHL) in the Public Medical Literature Analysis and Retrieval System Online (PubMed); Science Direct, Elsevier database; Scientific Electronic Library Online (SciELO); Latin American and Caribbean Health Sciences Literature (LILACS), as shown in Chart 2. After each search for descriptors/keywords/search strategies, the titles of the articles found were read, looking for words related to the researched topic, according to the PCC strategy (hospitals, conservation of natural resources, sustainability indicators, water consumption, health services waste, water consumption, environment, environmental health, sustainable development, environmental impact, energy consumption, conservation of natural resources, socio- environmental, green hospital). When the title was unclear, the abstract of the article was read.

### Selection of Studies

The step of data description and summarization was performed by two independent reviewers (DMG and MRCV), who read the abstracts and keywords in order to identify if the studies met the inclusion criteria. In case of any disagreement, a third reviewer was called to analyze and decide on whether or not to include the articles.

### Data Extraction

Data extraction (database, search criteria, journal, authors, year of publication, country of origin, title, digital object identifier – DOI, research question, objective, research approach, type of study, indicators of hospital sustainability used to measure the effectiveness of sustainability practices, evidence of reduced environmental impact, other observations) was performed and entered into a spreadsheet in Microsoft Office Excel, version 2016 for further analysis.

### Synthesis of Data

After filling out the worksheet, the two reviewers checked if their extractions were similar and any disagreement was sent to a third reviewer for analysis. After selecting the studies, the percentage of table data (year of publication, language, design and research approach) was calculated to present the results. Next, the data related to the hospital sustainability indicators used to measure the effectiveness of sustainability practices, and the evidence presented in these studies were analyzed.

Environmental sustainability indicators generally point to two groups of attention: optimization of resources (energy and water) and monitoring and mitigation of the impact of its activities (effluents, waste and emissions)^(11)^. For this reason, the articles were presented according to these categories in the results section. Another category was added for evidence of reduction of the socio-environmental impact in order to respond to the objective of the study.

## RESULTS

**Figure 1.**
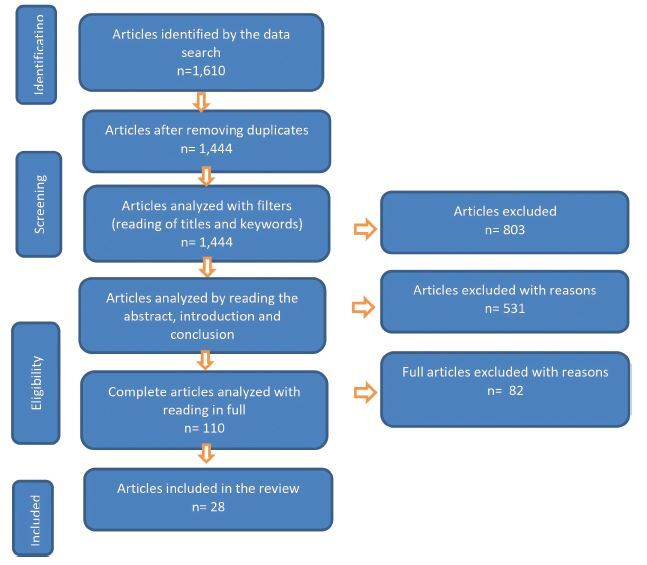
Diagram of the process of inclusion and exclusion of studies – Rio Grande, RS, Brazil, 2022.

In the data search, 1,513 studies were identified with descriptors and another 97 studies through other sources, totaling 1,610 studies. Of these, 166 were excluded because they were duplicated in databases. After reading and analyzing the title and abstract, 803 out of the 1,444 studies were excluded because they were not aligned with the object of study. Then, another 614 studies were excluded for other reasons. At the end of the selection, 28 articles remained, as shown in Figure 1.

Regarding the language of articles, 75% (21 articles) were published in English. Articles in Portuguese, German and Arabic languages represented 7.14% (two articles) of publications each, and 3.57% (one article) were published in Italian. Most articles (85.71%, corresponding to 24 articles) were quantitative and the remaining four articles (14.29%) were qualitative/quantitative.

The time interval of retrieved studies was between years 2012–2020; 25% of the articles (7) were published in 2012, 21.43% (6) in 2017, 10.71% (3) in 2015, and 14.29% (4) in 2016. In years 2013, 2014 and 2018, 7.14% of articles (2) were related to the research object, and between 2019 and 2020, one article for each year, which corresponds to 3.57%

**Chart 3.**
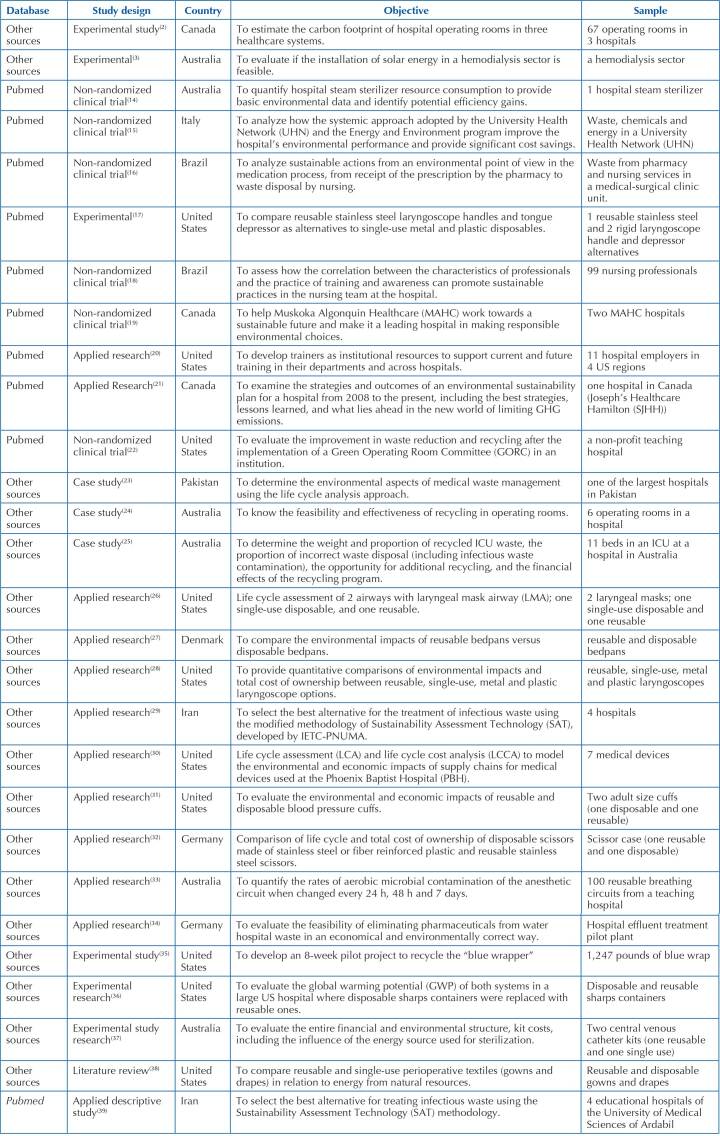
Characterization of publications retrieved in the search in electronic databases and search by references – Rio Grande, RS, Brazil, 2022 (n = 28).

With regard to the type of study, 12 of them (42.86%) were applied research, 21.43% (6 articles) were non-randomized clinical trials and experimental research studies, respectively; 3 (10.71%) were case studies and 1 (3.57%) was a literature review, as shown in Chart 3.

Nursing is present in these studies through its work, from choosing which material to use during a procedure to its disposal in the environment. However, nurses were the authors in only five studies (2 Brazilian studies)^(14,15)^, one from Canada^(15)^ and two from the United States of America^(16,17)^. The other authors were from the areas of biology^(2)^, biostatistics^(16)^, biochemistry^(17)^, geology^(18)^, chemistry^(19)^, medicine^(3,17,19–30)^, marketing^(20,23)^, engineering^(19,20,22–25,30–38)^ and administration^(17,20,22,39)^.

**Chart 4.**
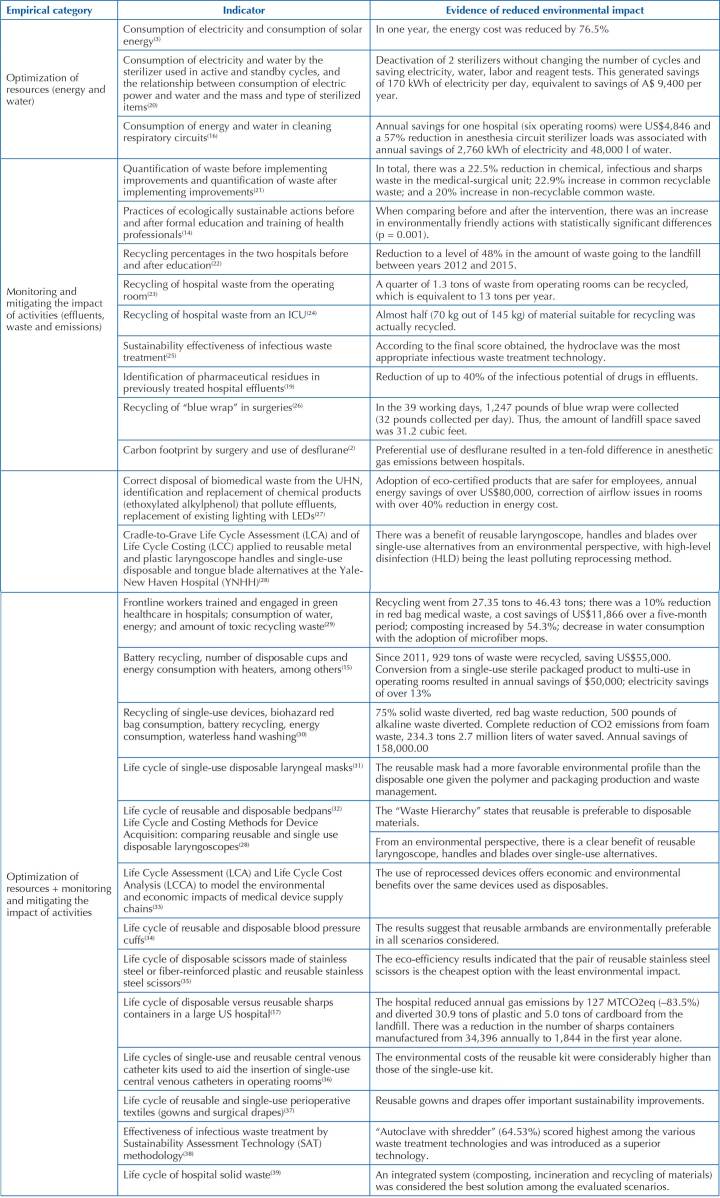
Empirical categories that emerged from the studies included in the scoping review – Rio Grande, RS, Brazil, 2022 (n = 28).

Environmental sustainability indicators generally point to two attention groups; optimization of resources (energy and water) and monitoring and mitigation of the impact of its activities (effluents, waste and emissions)^(11)^, such as an integrated system of composting, incineration and recycling of materials^(35)^. From the selection of articles in this scoping review emerged two empirical categories for indicators, and another category was added for evidence of reduced socio-environmental impact. They are explained in Chart 4.

## DISCUSSION

There is a significant environmental impact of activities involving the health sector. Therefore, it is necessary to know the reality of sustainability where the hospital is located. Indicators can be used for this purpose, as they allow managers to have a multidimensional view between the current state of sustainability of hospitals and their level of excellence. Through indicators, it is also possible to identify sustainability-related strengths and weaknesses of the hospital, enabling a more assertive definition of public policies^(8)^.

Many studies have been developed to improve the sustainable structure of the hospital, reduce energy and water consumption, and waste generation^(40)^. Regarding the optimization of resources (water and energy) in hospitals, indicators related to electric power consumption, water consumption and solar energy consumption were cited in the studies included in this review^(7,16,27)^.

With regard to water consumption, the hemodialysis sector is one of the most harmful in the health sector. It is estimated that patients who use this therapy for four hours a week consume an average of 500 liters of water per treatment and another 500 liters for sterilization, priming, pre- and post-hemodialysis^(41)^.

Hand washing in the operating room is another activity where water is wasted. The installation of a pedal-activated intermittent flow system can reduce waste by up to 14 liters of water per hand wash^(42)^.

In addition to damage to the environment, there is also unnecessary expense to hospitals. In a hospital in Australia, it was identified that the simple deactivation of two material sterilizers without changing the number of cycles and saving electricity, water, labor and reagent tests would generate a saving of 170 kWh of electricity per day, equivalent to savings of A$9,400 per year^(24)^.

A similar study in a hospital with six operating rooms identified that for a 57% reduction in the anesthesia circuit sterilizer loads, there was an annual saving of 2,760 kWh of electricity and 48,000 l of water. The use of photovoltaic solar energy powered by the sun is among the energy saving alternatives, as this an abundant source with inexhaustible incidence on the earth’s surface^(16)^.

A study conducted in Australia demonstrated that in one year of implantation of a solar panel in a hemodialysis service, the energy cost reduced by 76.5%, with a prediction of free service, including installation costs, in 7.7 years^(3)^.

As for monitoring and mitigating the impact of its activities (effluents, waste and emissions) in hospitals, the selected studies^(9,11,12,18,19,23,28,29,31,34,35)^ brought indicators related to the quantification of waste before and after interventions, recycling of hospital waste, effectiveness of infectious waste treatments, life cycle of solid waste, carbon footprint in surgeries and identification of pharmaceutical residues in effluents.

Improper solid waste handling at any stage of management processes can cause impacts and pollute water, soil and air, altering chemical, physical and microbiological environmental factors^(43)^. The National Solid Waste Policy was instituted in 2010 with the aim to preserve public health and environmental quality in the sense of non-generation, reduction, recycling and treatment of urban solid waste and waste from health services^(44)^.

A study developed in a hospital in Brazil in 2016 analyzed environmentally sustainable actions in the medication process from receipt of the prescription by the pharmacy until the disposal of waste by nursing professionals, and identified a 74.8% reduction in chemical, infectious and sharps after educational and routine interventions^(21)^. In another study, when comparing before-and-after a waste disposal educational intervention, an increase in ecologically correct actions was identified^(14)^.

Another concern of hospitals in relation to waste is the selection of more sustainable practices for treating infectious waste, with a view to reducing environmental risks and the spread of diseases^(38)^.

A study performed in Iran investigated the most sustainable alternative for treating infectious waste; hydroclave, autoclave with shredder, autoclave, central incineration and chemical treatment. Based on technical, economic, social and environmental aspects, the hydroclave was the most sustainable technique^(25)^.

In another study, also conducted in Iran, the “autoclave with shredder” obtained the highest score (64.53%) among the various waste treatment technologies and was introduced as a superior technology. However, the study points out that it is not mandatory to use the technology with the highest score. The specific conditions of each hospital in relation to environmental, technical, social and economic aspects should be taken into account^(38)^.

Recycling is another approach adopted in relation to hospital waste. The education of people involved in waste segregation is one of its most important points. Once more, the importance of awareness of the nursing team is emphasized, as this is usually the category with the largest number of people in a health service. A comparative study conducted in two hospitals in Canada between 2012 and 2015 showed a 48% reduction in waste that would go to the landfill after a continuing education program on recycling in these hospitals^(22)^.

Unfortunately, not all medical waste can be recycled given its infectious potential. A study performed in an intensive care unit (ICU) in a hospital in Canada showed that only 14% out of 28% of waste that could be recycled were effectively recycled given the cross infection of materials in the disposal^(24)^, demonstrating the importance of continuing education for workers in these services.

The operating room, like the ICU, is another part of the hospital with a large amount of infectious waste, but also with a large amount of packaging and wrappings that can be easily recycled. It is estimated that a quarter of every 1.3 tons of waste produced in the operating room can be recycled, which is equivalent to something around 13 tons per year^(23)^. The “blue wrap”, a number 5 polypropylene plastic material used in operating rooms to pack instrument trays, is another example of material that can be easily recycled and generate savings for the hospital, and reduce the space for waste in landfills^(26)^.

Another concern of hospitals in relation to sustainability concerns the release of pharmaceutical waste in effluents. They are excreted through human feces and urine, which, along with other waste, are transported to municipal water treatment plants, although these plants are not designed to remove these types of waste. A study conducted in Germany demonstrated that the use of ultraviolet irradiation (UV) is effective in the degradation of more persistent drugs in effluents, which reduces the environmental impact of their release^(19)^.

The other studies included in this scoping review^(8,10,13–15,20,21,24–26,30,32,33)^ deal with the optimization of resources (water and energy) combined with the monitoring and mitigation of the impact of their activities (effluents, waste and emissions) as a way to reduce costs and reduce the environmental impact at the same time. The indicators of these studies point to the disposal of biomedical waste, replacement of chemical products that pollute effluents, replacement of old lighting with light- emitting diode (LED) lamps, assessment of life cycles, training of health workers in sustainability actions, recycling of materials, conscious consumption and carbon footprints.

The life cycle of an activity or product evaluates the environment, the potential impacts of its products and processes around its life cycle, from the extraction of the raw material (cradle), its production, its use and the end of its life (grave)^(44)^. This makes it possible to identify opportunities for improving environmental performance at each stage of the life cycle^(45)^. On the other hand, the carbon footprint is a quantitative measure of direct and indirect GHG emissions related to a process, product, institution or industry. It is expressed in equivalent mass (kilograms) of carbon dioxide (CO2) released into the environment^(7)^.

A study carried out in Pakistan between 2014 and 2015 evaluated the life cycle (regarding GHG emission) of a ton of disposable hospital solid waste from its transport until treatment, landfill disposal, incineration, composting and recycling of materials. The most sustainable among the alternatives was the performance of an integrated system between composting, incineration and recycling^(18)^.

A study evaluating the life cycle and costing methods for the acquisition of reusable and disposable laryngoscopes was carried out in the United States. The result was that reusable handles of laryngoscopes had advantages in terms of cost and environmental sustainability compared to disposable handles^(28)^.

In 2017, another study evaluating the carbon footprint of three operating rooms in different hospitals was carried out in the United States. It was identified that the use of the anesthetic desflurane increased the carbon footprint in hospitals by 10 times, including the increase in energy consumption^(2)^.

There are several published studies evaluating the life cycle of products. One of them analyzed the life cycle of reusable and single-use laryngeal masks and described that reusable masks are less harmful to the environment^(31)^. Another similar study with bedpans identified that reusable bedpans are preferable to disposable ones^(32)^. Other studies with laryngoscopes^(45)^, pressure cuffs^(34)^, scissors^(35)^, sharps containers^(17)^ and perioperative textiles (surgical gowns and drapes)^(37)^ also correlated the use of reusable materials with less damage to the environment compared to single-use materials. However, there is a caveat; the excessive use of reusable materials correlates with a greater environmental impact than disposable materials^(33)^.

Only one study that evaluated the life cycle of central venous catheter kits was in agreement with all the previously cited life cycle studies, reporting that reusable kits had higher environmental costs than disposable kits. This is a result of the use of electric power from brown coal in the studied hospital, which is directly related to an increased environmental impact. This study reinforces that the use of this method is reliable and helps hospital managers in decisions about sustainability. However, the particularities of each hospital must be taken into account^(36)^.

Regarding the recycling of materials, a hospital in the United States bet on a program to engage frontline workers to reduce the consumption of water, energy and toxic waste. The result was an increase of 19.08 tons in recycled materials and a 10% decrease in infectious waste. There was also a reduction in water consumption with the adoption of microfiber mops and the use of smaller buckets by cleaning staff; reduction of energy consumption with the practice of turning off computer monitors that were not in use and replacement of cleaning products with toxic components with ecologically acceptable products^(29)^.

Another study at a hospital in Canada also adopted eco- certified products that are safer for staff and the environment, and managed to save over US$80,000 in energy in one year just by correcting airflow problems in inpatient rooms, which generated savings of 40%^(27)^.

A US hospital created an operating room committee to adopt sustainable practices in surgery, and they were able to reduce 12,860 pounds of solid waste by recycling single-use products; foam pads were replaced with reusable gel pads, saving over $50,000 a year. Batteries were discarded, recovered and distributed to the hospital or donated to charity (annual savings of US$9,000)^(30)^.

The initiative to turn off all lights and equipment of anesthesia and the operating room that were not in use resulted in savings of US$33,000 and 234.3 metric tons of CO2 emissions reduced per year. Replacing soap with an alcohol-based waterless exfoliant has shown savings of 2.7 million liters of water annually^(30)^.

Strategies used by another hospital in Canada included recycling 929 tons of waste in three years, which generated savings of US$55,000. Another action was the change from single-use materials to multi-use in operating rooms, which resulted in annual savings of US$50,000. In addition, energy savings of over 13% were achieved just by turning the heater off. Batteries began to be reused and disposable cups were replaced by reusable ones^(15)^.

Limitations of the present review are related to the nature of the review itself, mainly in the indication of subsidies for the formulation of policies, since its proposal is to provide an overview of the socio-environmental indicators studied, which may not be sufficient to structure the guiding elements for the decision- making process in different instances, such as in the management and governance of the organization. Furthermore, no procedures were used to evaluate the evidence found. Therefore, the deepening of findings through other review studies is suggested. The relevance of this study is corroborated in view of the synthesized content about hospital sustainability indicators and reduction of socio-environmental impacts.

The advances achieved with this study were to know which indicators and evidence are being more used in hospitals and help to reduce the socio-environmental impact, so these can serve as reference for other hospitals, whether by repeating successful actions or testing new technologies to reach better financial, social and environmental results.

## CONCLUSIONS

The present study made it possible to synthesize knowledge about hospital sustainability indicators and evidence on the reduction of socio-environmental impact. The indicators of this review pointed to the optimization of water and energy resources and the monitoring and mitigation of the impact of its activities (effluents, waste and emissions).

Resource optimization indicators (energy and water) were related to the use of sterilizers, the processing and sterilization of medical and hospital products, and energy savings through the solar energy system. Evidence of reduced socio- environmental impacts indicated energy savings with the use of fewer sterilizers without compromising the cycles, reduction in energy and water consumption by using larger loads of products without compromising their sterilization, and savings of 76% in spending of electric power by implanting a solar panel in a health service.

On the other hand, the indicators for monitoring and mitigating the impact of its activities (effluents, waste and emissions) were related to the quantification of waste before and after the implementation of improvements, practices of ecologically sustainable actions and recycling before and after formal education, effectiveness of sustainability of the treatment of infectious waste and hospital effluents, carbon footprint and life cycles of surgeries and medical-hospital supplies.

Evidence of the reduction of socio-environmental impacts in effluents, waste and emissions include a reduction in the amount of infectious and sharps waste and an increase in the amount of recyclable waste, identification of more appropriate and less harmful technologies for the environment in the treatment of infectious waste, reduction of carbon emissions in surgeries and choice of disposable or single-use materials according to lower environmental impact, such as pressure measurement cuffs and laryngoscopes.

There are several up-to-date studies on the subject focusing on optimizing resources and monitoring and mitigating the impact of hospital activities. The results of the indicators of these studies show good evidence of reduced socio-environmental impact, which can help hospital managers to select the best practices and apply them in their institutions with a view to reducing socio-environmental impacts in hospitals.
